# Health in War Industries

**Published:** 1919-03-15

**Authors:** 


### Health in War Industries.

At a meeting of the Royal Statistical Society, Captain M. Greenwood, in a paper on "Problems of Industrial Organisation," gave account of the recent labour wastage in factories employing women. In very few were more than 80 per cent. of the entrants still at work at the end of three months, while in some the percentage fell as low as 65-70. Fortunately, this heavy wastage was greatly reduced by effective welfare departments, and the system of canteen and hostel feeding furnished statistics proving that during the most anxious period of the war large numbers of munition workers received adequate diets. The figures on relation between hours of labour and output proved an oft-disputed fact that frequently a reduction of hours was attended by not only a relative but an absolute increase in productive power and health. An instance is given where an increase of 8 per cent. was found in the output when the working periods were reduced by more than ten hours a week. Similarly, proof was obtained that length of hours and temperature had a bad influence upon youthful physique and the frequency of all kinds of accidents, allowing the introduction of many reforms in these matters. The paper suggests a field of intensely interesting research in the medical and psychological problems of industrialism.

